# Utility of Dried Blood Spots for the Diagnosis of Congenital Cytomegaloviruses within the First 21 Days of Life in a Single Center

**DOI:** 10.3390/ijns9030044

**Published:** 2023-08-04

**Authors:** Ana Del Valle Penella, Jerry Miller, Ryan Rochat, Gail Demmler-Harrison

**Affiliations:** Department of Pediatrics, Division of Infectious Disease, Baylor College of Medicine, Houston, TX 77030, USA; delvalleanapaula@gmail.com (A.D.V.P.); jamiller@texaschildrens.org (J.M.); rochat@bcm.edu (R.R.)

**Keywords:** congenital CMV, diagnosis of congenital CMV, newborn screening for congenital CMV, Dried Blood Spot, hearing loss

## Abstract

In this retrospective study, we aimed to evaluate the performance of dried-blood-spot (DBS) testing as a diagnostic method for the congenital cytomegalovirus (cCMV). We reviewed the medical records and DBS test results of 89 patients who had also undergone diagnostic cCMV testing within the first 21 days of life. The DBS test had a sensitivity of 83.9%, a specificity of 100%, a positive predictive value of 100%, and a negative predictive value of 73%. Patients with a true-positive DBS had a higher median level of CMV in blood according to PCR than those with a false-negative result. Additionally, all patients with cCMV and hearing loss had a positive DBS test, with higher median viremia levels observed in those with hearing loss compared to those without a CMV PCR blood test. These results suggest that DBS-based testing is useful in the diagnosis of cCMV, and its performance may be related to levels of CMV viremia. DBS testing accurately identified those patients with congenital/early onset hearing loss and those at risk of developing late-onset hearing loss.

## 1. Introduction

The congenital cytomegalovirus (cCMV) remains the most common congenital infection in the United States and developed countries, as well as the leading cause of non-genetic hearing loss and a significant cause of neurodevelopmental delays [1,2]. Despite its frequency, cCMV is an underdiagnosed condition, because the majority of those affected by it are asymptomatic at birth. Further compounding the complexity, a significant proportion of asymptomatic newborns with cCMV will experience later-onset progressive sensorineural hearing loss (SNHL) [3]. Moreover, those newborns with cCMV who are symptomatic at birth often have only subtle symptoms that can be missed, or they may experience delayed symptoms. Further complicating the diagnosis of cCMV is the strict diagnostic window of the first 21 days of life (DOLs) [4,5,6,7].

There is growing interest in finding a diagnostic method that can be utilized as a universal screening method for all newborns to increase early detection and interventions, improve outcomes, and avoid an expensive diagnostic odyssey and unnecessary testing [8,9]. Traditional testing for cCMV in newborns currently relies on the detection of CMV in saliva, urine, whole blood, or plasma via cell culture assays or molecular PCR testing. The detection of CMV DNA using PCR assay techniques performed on dried blood spots (DBSs) in newborns has been used for the retrospective diagnosis of cCMV, as well as a newborn screening method for cCMV [6,10,11,12,13,14,15].

The aim of this study was to retrospectively collect and analyze the utility of the detection of CMV DNA using PCR in DBS for the diagnosis of cCMV in patients suspected of having congenital CMV who were tested via traditional methods of detecting CMV in saliva, urine, or blood plasma using CMV cell culture or CMV PCR within the first 21 DOLs at Texas Children’s Hospital and relate the results to cCMV disease classification and SNHL.

## 2. Materials and Methods

### 2.1. Study Population and Design

**Figure 1:**
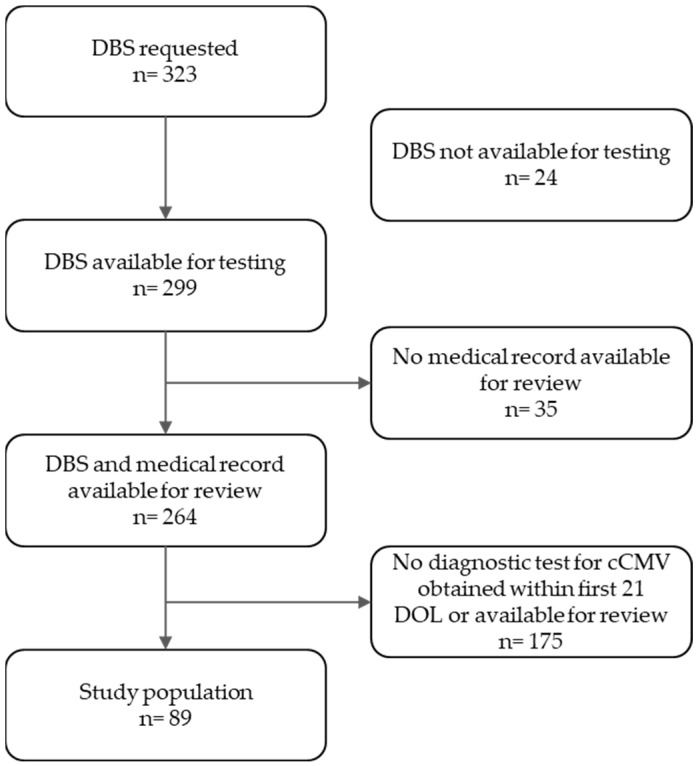
Inclusion and exclusion criteria of the study population.

In this single-center retrospective study, all patients (*n* = 323) with a request for DBS testing at Texas Children’s Hospital between 2013 and 2021 were reviewed. Those with a DBS available for testing obtained within the first 21 DOLs, a medical record at our institution, and at least one diagnostic test for cCMV (urine cell culture, urine PCR, blood PCR and/or saliva culture, or PCR for CMV) obtained within the first 21 days of life (DOLs) available for review were included in the study (*n* = 89) Figure 1.

### 2.2. DBS Retrieval and Storage

DBSs were requested from the Texas State Health Department using the parent consent form for the release of DSHS newborn screening specimens and the request for the release of an individual’s newborn screening specimens (F14-13561) (http://www.dshs.state.tx.us/lab/newbornscreening.shtm; accessed on 29 July 2023) and sent to our institution. The retrieved DBS were then sent to the Center for Disease Control (CDC) for CMV DNA extraction and CMV PCR testing.

The DBS were stored at air-conditioned room temperature at the DSHS newborn screening laboratory until retrieved, and they were mailed by overnight courier to the CMV Laboratory at Texas Children’s Hospital. Upon receipt at the Texas Children’s Hospital CMV Laboratory, the DBSs were placed in a plastic zip lock bag with a desiccant and a humidity indicator card and stored at either −80 °C or cooled at air-conditioned room temperature, until then they were mailed via overnight courier to the CDC laboratory. DBS were then tested within one week of receipt at the CDC laboratory.

### 2.3. CMV DNA Extraction and PCR Testing

DBS CMV DNA extraction and PCR testing were performed at the CDC with the methodology published by Dollard et al. The sensitivity of the assay was 5 copies of the CMV/PCR reaction [11]. The CDC was blinded to all diagnostic and clinical information on the patient for whom the DBS was tested.

Results were reported as positive, negative, or equivocal. An equivocal result was reported when the cycle threshold value fell in between the values of established positive and negative results. In some cases, the same DBS specimen, using a different whole punch from the specimen, was extracted and tested again, which usually resolved the result as positive or negative. A final equivocal result was considered negative for analysis. Quantitative values for CMV were provided for those with a positive DBS.

### 2.4. Data Collection

All clinical data were retrieved from the electronic medical record (EMR) and recorded using REDCap hosted at Baylor College of Medicine. The EMR was reviewed for demographic data, antenatal history, symptoms present at birth, outcomes, method of diagnosis of cCMV, and CMV viremia levels. CMV viremia was determined using plasma PCR and documented as IU/mL. For those results reported as copies/mL, a conversion of 1 IU/mL per 0.53 copies/mL of CMV was utilized as per the Viracor-Eurofins internal calibration of the World Health Organization standard. In rare cases, patients’ plasma PCRs were carried out at local hospitals or other reference laboratories.

### 2.5. Data Analysis

The first DBS results, collected within the first 2 days of life, were compared to the diagnostic results of patients whose diagnoses were made via traditional testing with at least one accepted method (urine culture, urine PCR, blood PCR and/or saliva culture, or PCR) obtained within the first 21 DOLs. Sensitivity, specificity, positive predictive value, and negative predictive value were calculated. Statistical analysis was performed using SPSS Version 28.0 (IBM SPSS, Inc., Armonk, NY, USA). A Wilcoxon Rank Sum Test was utilized to compare independent samples.

### 2.6. Definitions

- Confirmed cCMV (CcCMV): infant with virological confirmation of CMV infection via urine culture, urine PCR, and blood PCR and/or saliva culture within the first 21 DOLs.
- Not congenital CMV (NcCMV): infant with a negative confirmatory test within the first 21 DOLs.
- cCMV disease classification, as published by Kimberlin and colleagues, with minor changes based on discussions of the International cCMV Recommendations Group [7]:
- Asymptomatic cCMV: no apparent abnormalities to suggest cCMV and normal hearing.
- Asymptomatic cCMV with isolated SNHL: no apparent abnormalities to suggest cCMV, but SNHL present (≥21 decibels)
- Mild symptomatic cCMV: one or two isolated manifestations of cCMV infection that are mild or transient (i.e., mild hepatomegaly or a single measurement of low platelet count).
- Moderate to severe symptomatic cCMV: multiple manifestations attributable to cCMV or Central Nervous System (CNS) involvement such as microcephaly, radiographic abnormalities consistent with CMV CNS disease (ventriculomegaly, intracerebral calcifications, periventricular echogenicity, cortical or cerebellar manifestations), abnormal cerebrospinal fluid (CSF) indices for age, chorioretinitis, SNHL, or the detection of CMV DNA in CSF.
- We categorized SNHL as follows:
  - Congenital: infants diagnosed using auditory brainstem response (ABR) in one or both ears within the first month of life or a failed hearing screen with a diagnostic ABR within the first year of life.
  - Early onset: a passed hearing screen with an abnormal ABR assessment from ≥1 month to 12 months of life.
  - Delayed onset: detected after ≥1 assessments with normal hearing after 12 months of life.

## 3. Results

Eighty-nine infants who underwent evaluation for cCMV at Texas Children’s Hospital and who had an available traditional urine, saliva, or blood plasma cell culture or PCR test performed within the first 21 DOLs for cCMV infection had their DBS retrieved and tested. Most infants were non-Hispanic (74%), white (68%), and born at full term (74%), with the most common reason for testing being signs and symptoms suggestive of cCMV (61%), followed by having a failed hearing screen (36%); more than one reason for screening per patient was documented in some cases. Demographics for the study population are presented in Table 1.

**Figure 2:**
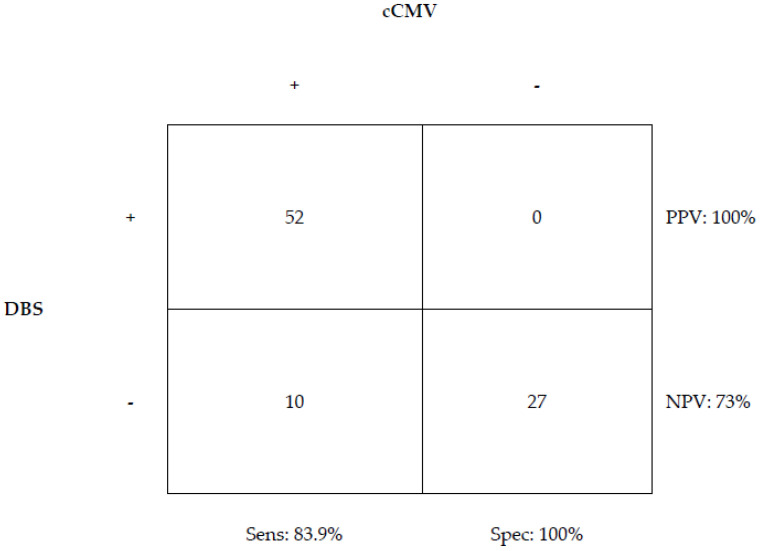
Sensitivity and specificity of DBS when compared to patients with a diagnosis of cCMV using traditional testing.

Of the 89 infants tested, 62 (70%) had a final diagnosis of CcCMV established via conventional testing, and 27 (30%) were classified as NcCMV. Among the 62 infants with CcCMV, 52 had a positive DBS and 10 had a negative result. DBS had a sensitivity of 83.9% (52 out of 62) and a specificity of 100% (0 out of 27), a positive predictive value of 100%, and a negative predictive value of 73% (Figure 2). The clinical characteristics of those patients with a false-negative result are presented in Table 2.

**Figure 3:**
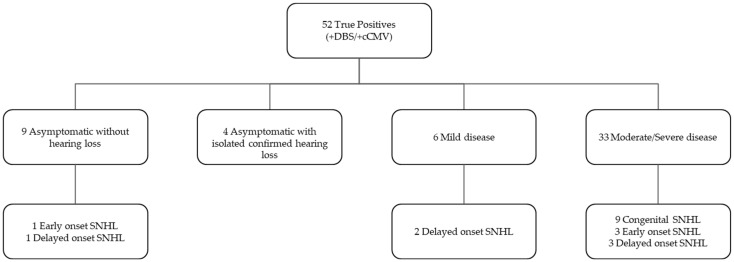
Clinical characteristics of patients with a true-positive DBS result.

Out of the 62 infants with CcCMV, 52 had a positive DBS result, 9 (17%) had asymptomatic cCMV without hearing loss, 4 (8%) had asymptomatic cCMV with isolated hearing loss, 6 (12%) had mild symptomatic cCMV, and 33 (63%) had moderate to severe symptomatic cCMV. A total of 10 infants who did not have congenital hearing loss went on to develop SNHL later in life (Figure 3).

**Figure 4:**
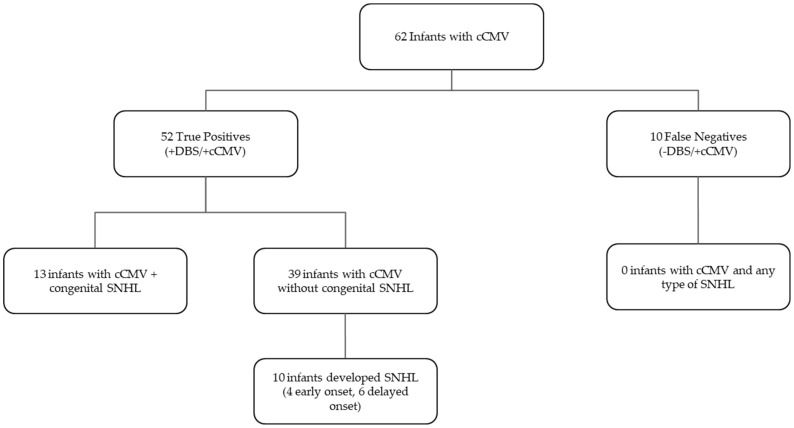
Patients with confirmed cCMV and SNHL at any time were all detected using DBS testing.

All infants diagnosed with congenital hearing loss and CcCMV had a positive DBS result. Equally, all infants with early onset and late onset hearing loss had a positive DBS result (Figure 4).

A viral load from the first DBS sample, collected within first 2 days of life, was reported for 51 DBS with a median level of 3073 IU/mL (IQR, 1383–7956 IU/mL). No significant difference was found when comparing DBS viral load between infants with and without hearing loss, as well as among different subgroups (Table 3). No significant difference in viral load was noted between different classifications of symptomatic cCMV.

**Figure 5:**
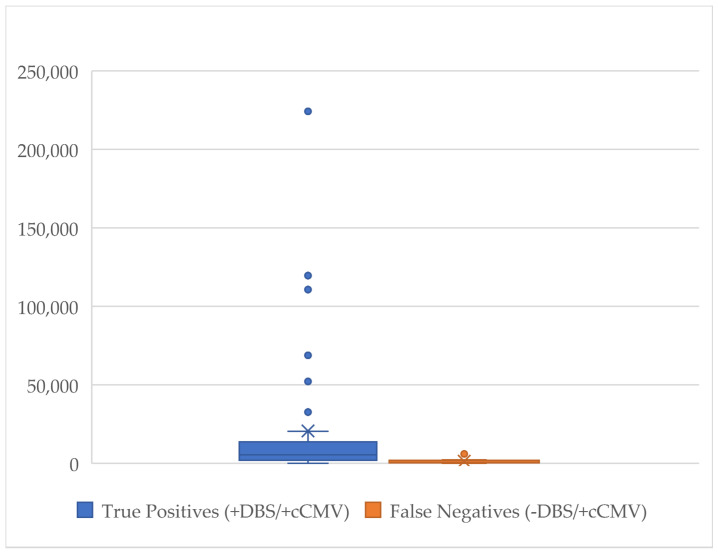
Comparison of viremia levels (IU/mL) in patients with a blood CMV qPCR obtained within first 21 DOLs with a true-positive DBS vs. false-negative DBS.

Among the 62 patients with CcCMV, 46 had CMV PCR blood obtained clinically within the first 21 DOLs. For these patients, the median viremia level of those with a true-positive DBS (*n* = 38) was found to be significantly higher compared to those patients with a false-negative DBS (*n* = 8): 11,237 IU/mL vs. 535 IU/mL, *p* = 0.003 (Figure 5).

Infants with CcCMV and any type of SNHL (*n* =18) also had a significantly higher level of median viremia level according to CMV PCR blood when compared to those infants with CcCMV and no SNHL (*n* = 28): 11,406 IU/mL vs. 4867 IU/mL, *p* = 0.003 (Table 3).

When patients whose infections were confirmed within the first 21 days of life were divided into subcategories of hearing loss, those with congenital hearing loss had a significant difference in their median levels of blood CMV when compared to those with no hearing loss: 15,001 IU/mL vs. 2405 IU/mL, *p* = 0.007. A significant difference was also observed when infants with congenital and early onset hearing loss were grouped and compared to those without hearing loss: 19,065 IU/mL vs. 2405 IU/mL, *p* = 0.001. No significant difference was found for infants who went on to develop late-onset hearing loss (Table 3). No significant difference in blood CMV levels was noted between different grades of symptomatology.

Thirteen infants had a discordant result between their two DBSs collected within the first 21 DOLs (Table 4). Out of the 12 infants with an initial positive result and a second DBS that was either equivocal or negative, 8 (67%) were started on antiviral treatment prior to the collection of the 2nd DBS. Only one infant had an initial negative DBS test collected within the first 2 days of life, which was followed by a second DBS obtained within the first 21 DOLs with a positive result; this infant’s mother received treatment during pregnancy with a high dose of valacyclovir.

## 4. Discussion

In this retrospective study, the DBS had a sensitivity of 83.9% when utilized for the diagnosis of cCMV and a specificity of 100%. These findings are higher than the 73% and 77% sensitivity reported when it was utilized as a screening method in the laboratories at the University of Minnesota and CDC, respectively [11]. In contrast, Leurez-Ville et al. reported a higher sensitivity of 95% and 96.9%, with a similar specificity of 98.5 and 99%. Possible explanations for the variation in performance of the test using DBS in these studies include differences in (1) DBS size, as a larger DBS size could lead to increased sensitivity; (2) PCR methods; and (3) characteristics of the populations being studied [13].

No significant difference in viral load of the first collected DBS was found in the different cCMV disease classifications or the different types of SNHL in our study population. This finding differs from what was previously reported by Walter et al., who found that 25 patients with cCMV and SNHL had a mean DBS log viral load that was significantly higher than that of the nine infants with cCMV and without SNHL [16]. Similarly, Leurez-Ville et al. reported significantly higher DBS viral loads in symptomatic infants compared to those who were asymptomatic at birth when using one CMV PCR assay, but they found no significant difference when utilizing their second assay [13].

It has been reported that the level of clinical viremia in samples collected outside of the DBS could be correlated with the risk for SNHL or the development of late-onset disease [16,17,18]. In our study, a significant difference was also found between the median level of clinical viremia in those infants with cCMV and any type of SNHL compared to patients with cCMV and without SNHL. The different results associating SNHL with viral levels, between the first collected DBS viral load and clinical viremia levels collected within the first 21 days of life, may be due to the timing of the collections, since viremia levels may escalate after birth.

Newborns with cCMV may not be diagnosed at birth and may go through a diagnostic odyssey before the diagnosis is established. A recent study looking at the retrospective diagnosis of children with cCMV via the utilization of DBS found that 19/436 (4.4%) of the children evaluated at their clinic had a delayed diagnosis of cCMV with multiple missed opportunities for the diagnosis of cCMV in both symptomatic and asymptomatic patients [6]. In another study, DBS was found to be useful in the retrospective diagnosis of congenital CMV in children suspected of having congenital CMV past the diagnostic window of 21 days [19]. In our population, all infants with congenital/early-onset SNHL and late-onset SNHL were accurately diagnosed using DBS. If DBS can accurately identify all those infants who are born with congenital/early-onset hearing loss or who are at risk of developing it or other late-onset sequelae, it would be appropriate to establish this method as a universal screening tool to provide timely interventions, prevent sequelae, and possibly provide cost savings to the healthcare system by avoiding the diagnostic odyssey often embarked upon by patients to determine the cause of their hearing loss [20,21].

Antiviral treatment reduces CMV viremia levels [22]. Therefore, antiviral treatment may be a contributor to false-negative results in the second DBS seen in our study. It is also possible that prenatal treatment contributed to the false-negative result in the first DBS for the infant whose mother received a high dose of valacyclovir treatment during pregnancy.

There are several limitations to our study. Since the population selected for the testing of their DBS comprised patients who were already undergoing evaluation for suspected cCMV, the positive predictive value calculated may not be extrapolated to the general population since the prevalence of cCMV was higher in our population. Because the testing of DBS was conducted between 2013 and 2021, there could be some variation in the methodology for DNA extraction and PCR methodology over the years. Finally, although the collection of CMV blood PCR samples was carried out within the first 21 DOLs, the exact day of collection varied from infant to infant, and the date of collection was therefore not uniform.

## 5. Conclusions

DBS was accurate in the diagnosis of cCMV when compared with traditional diagnostic methods for the detection of CMV in urine, saliva, and blood plasma collected within the first 21 days of life, and its performance may be related to the level of CMV viremia at birth. DBS appeared to accurately identify patients with congenital/early-onset hearing loss and infants who will develop later-onset hearing loss. In this study, the level of viral load of the DBS did not appear to differ significantly between clinical subgroups of infants with cCMV.

## Figures and Tables

**Table 1:** Patient Demographics.

Characteristic		All Patients, *n* = 89	
**Gender**	Male	42	47%
	Female	47	53%
**Ethnicity**	Hispanic	22	25%
	Non–Hispanic	66	74%
	Unknown	1	1%
**Race**	White	61	69%
	Asian	5	6%
	Black/African-American	21	24%
	Other	0	0%
	Unknown	2	2%
**Gestational age (weeks)**	>40	3	3%
	37–40	66	74%
	34–36	12	13%
	<34	8	9%
**Birth weight (g)**	≥2500	52	58%
	1500–2500	28	31%
	<1500	6	7%
	Unknown	3	3%
**Maternal age**	≤20	16	18%
	21–30	48	54%
	31–40	23	26%
	Unknown	2	2%
**History of IUGR**	Reported	33	37%
**Reason for testing**	History of maternal infection	27	30%
	Failed hearing screen	32	36%
	Signs and symptoms suggestive of cCMV	54	61%
**cCMV classification**	Asymtomatic at birth	11	12%
	Asymptomatic with isolated hearing loss	4	5%
	Mild disease	10	11%
	Moderate/severe disease	37	42%
	Not cCMV	27	30%

IUGR = intrauterine growth restriction.

**Table 2:** Clinical characteristics of infants with cCMV and a false-negative 1st DBS result.

Subject	Disease Severity	Hearing Loss?	Long-Term Sequelae	Testing Done for cCMV	CMV qPCR Plasma (IU/mL)
38	M	No	None	BP, UC	<471
63	M	No	None	BP, UC	<471
131	M/S	No	Learning delays, growth failure	BP, UP	2400
162	A	No	None	BP, UP	0
189	A	No	None	BP, UP	598
213	M	No	None	BP, UP	ND
221	M	No	None	UP	ND
254	M/S	No	Hypertonia	BP, UP	<471
302 *	M/S	No	None	BP, UP	6040
323	M/S	No	None	BP, UP	1099

* Mother received treatment during pregnancy, 2nd DBS was positive. A = asymptomatic without hearing loss, M = mild disease, M/S = moderate to severe disease, ND = not done. BP = Blood PCR, UC = Urine culture, UP = Urine PCR.

**Table 3:** Comparison of median viral load of 1st DBS and blood CMV PCR based on SNHL classification.

Clinical Characteristic	N	DBS Median Viral Load (IU/mL)	*p*	N	CMV in Blood Median Viral Load (IU/mL)	*p*
No SNHL	29	2758 (IQR 1479–8759)		28	2405 (IQR 723–5974)	
SNHL present	22	3421 (IQR 859–7292)	0.662	18	11,406 (IQR 2863–56,382)	0.003
Absent vs. congenital SNHL	12	2885 (IQR 1725–7723)	0.864	12	15,001 (IQR 2541–100,304)	0.007
Absent vs. early onset SNHL	4	5575 (IQR 4510–27016)	0.295	2	42,490 (IQR not applicable)	0.020
Absent vs. late onset SNHL	6	824 (IQR 545–8513)	0.115	4	5628 (IQR 1237–9558)	0.425
Absent vs. congenital + early onset SNHL	16	4046 (IQR 2381–7723)	0.758	14	19,065 (IQR 2863–79,347)	0.001

**Table 4:** Discordant results between the 1st and 2nd DBSs obtained within the first 21 DOLs.

Subject	1st DBS	DBS qPCR (IU/mL)	2nd DBS	DBS qPCR (IU/mL)	Traditional Testing	Disease Severity	Hearing Loss Onset	CMV qPCR Plasma (IU/mL)	Antiviral Treatment	Started Prior to Collecting 2nd DBS?
302 *	−	NA	+	7870	+	M/S	No	6040	Yes	Yes
25	+	2543	−	NA	+	M/S	Congenital	17,547	Yes	Yes
59	+	689	−	NA	+	M/S	Congenital	497	Yes	Yes
95	+	2606	−	NA	+	M/S	No	1700	Yes	Yes
96	+	3597	E	NA	+	M/S	No	6800	Yes	Yes
103	+	920	−	NA	+	M/S	No	13,600	Yes	Yes
142	+	603	−	NA	+	M/S	Delayed	7160	Yes	Unk
205	+	766	−	NA	+	M/S	No	2767	Yes	Yes
207	+	369	−	NA	+	M/S	Delayed	4096	Yes	No
222	+	35,350	−	NA	+	M/S	No	2409	Yes	Yes
241	+	1719	E	NA	+	M/S	No	5774	Yes	Yes
242	+	1722	−	NA	+	M/S	No	1976	Yes	Unk
311	+	846	−	NA	+	A	No	NA	No	NA

* Mother received treatment during pregnancy. NA = not applicable, E = equivocal, Unk = unknown. A = asymptomatic w/o hearing loss, M = mild disease, M/S = moderate to severe disease.

## Data Availability

The data presented in this study are available on request from the corresponding author. The data are not publicly available due to privacy reasons.
